# How to Precisely Open the Internal Auditory Canal for Resection of Vestibular Schwannoma *via* the Retrosigmoid Approach

**DOI:** 10.3389/fsurg.2022.889402

**Published:** 2022-06-28

**Authors:** Chenguang Jia, Chengshi Xu, Mengyang Wang, Jincao Chen

**Affiliations:** ^1^ Department of neurosurgery, Zhongnan Hospital, Wuhan University, Wuhan, China; ^2^ Brain Research Center, Zhongnan Hospital, Wuhan University, Wuhan, China

**Keywords:** fusion image, endoscope, vestibular schwannoma, retrosigmoid approach, navigation

## Abstract

**Objective:**

The aim of this study was to investigate how to precisely expose the intrameatal portion of vestibular schwannomas (VSs) without damaging the labyrinth.

**Methods:**

This was a retrospective study of patients who had undergone retrosigmoid resection of a VS in our institution from April 2018 to December 2021. The patients were divided into microsurgery (MS) and navigation endoscopic-assisted (combined surgery, CS) groups and the effects of image guidance and endoscopy evaluated. The tumors in the CS group were then divided into medial and lateral types by fusion imaging and the differences between the two types analyzed.

**Results:**

Data of 84 patients were analyzed. Residual tumor was detected by postoperative MRI at the fundus of the internal auditory canal in 5 of the 31 patients in the MS group and 1 of the 53 in the CS group. The labyrinth was damaged in four patients in the MS group but was not damaged in any of the CS group patients. The CS group included 29 lateral type and 24 medial type schwannomas. Endoscopic-assisted resection of residual tumor in the IAC was performed significantly more often on medial than on lateral tumors.

**Conclusion:**

Navigation and endoscopy are useful in assisting the exposure of the intrameatal portion of VSs. Preoperative MRI/CT fusion imaging is helpful in preoperative evaluation and surgical planning in patients undergoing VS surgery. Tumors of the medial type require endoscopic assistance for resection.

## Introduction

Vestibular schwannoma (VS) resection *via* the retrosigmoid approach (RS) remains challenging. Although this approach provides excellent visualization, access to the fundus of the internal auditory canal (IAC) is limited (1–5). Removal of the posterior canal wall is essential to expose the portion of the tumor residing in the IAC (2, 3, 6–11). In some cases, microscopic exposure of the tumor is incomplete, even with excessive traction of the cerebellum or extensive drilling of the posterior wall of IAC (12). Use of an endoscope may solve this problem (13–16). However, endoscopic tumor resection is a skill set that is not necessarily learned quickly, nor are microscopic skills easily transferable to the technique (17). Furthermore, numerous reports have indicated that few tumors have a remnant at the fundus of the IAC that cannot be seen with the operative microscope (15, 18–22). In the past few years, we have performed image-guided microsurgery with endoscopic assistance for patients with vestibular schwannoma. Magnetic resonance imaging (MRI) and computed tomography (CT) fusion images are used for intraoperative navigation, as either modality alone provides inadequate clinical information. Together, they are complementary, and their data can be easily and accurately integrated with the navigation system software (23, 24). The aim of this study was to investigate how to precisely expose the intrameatal portion of VSs without damaging the labyrinth.

## Methods

### Patient Cohort

The cohort of this retrospective study comprised consecutive patients who had undergone RS resection of VS in our institution from April 2018 to December 2021. Patients with recurrent tumors, a history of radiation therapy, high jugular bulbar, and neurofibromatosis type 2 were excluded. Patients whose surgery was performed with the assistance of a microscope only were defined as the microsurgical group (MS group), whereas those whose surgeries were assisted by navigation and endoscopy were defined as the combined surgical group (CS group). Clinical data regarding patient age, sex, clinical presentation, neurological examination, neuroimaging, surgical findings, tumor size, and treatment outcomes were recorded and analyzed. The study was approved by the Medical Ethics Committee of Zhongnan Hospital of Wuhan University (2021037 K). Patients underwent routine follow-up every 3 months for 1 year and then yearly thereafter.

### Case Classification

**Figure 1:**
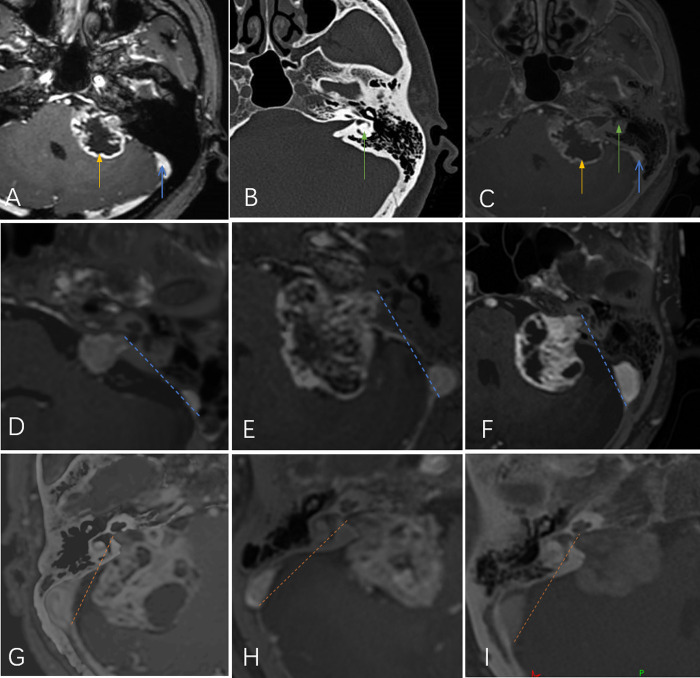
(**A**) Magnetic resonance imaging shows the tumor (yellow arrow) and sigmoid sinus (blue arrow). (**B**) Computed tomography shows the labyrinth (green arrow). (**C**) The fused image shows the positions of all three simultaneously. The line between the outermost edge of the tumor and the medial side of the sigmoid sinus was defined as the lateral safe line (LSL; yellow- or blue-dotted line). Tumors were classified according to the relationship between the LSL and the labyrinth: lateral type (**D,E,F**) and medial type (**G,H,I**).

Schwannomas in the CS group were further classified into medial and lateral types as follows: Digital imaging and communications in medicine (DICOM) MRI and CT files were imported into the navigation system station (Medtronic Planning Station S7, Louisville, KY, USA). StealthMerge® system software (Medtronic, Dublin, Ireland) was used to automatically fuse the images. After adjustment, the outline of the tumor and posterior wall of the IAC could be clearly displayed. The line between the outermost edge of the tumor and the medial side of the sigmoid sinus was defined as the lateral safe line (LSL). Cases were classified as medial or lateral according to the relationship between the LSL and the labyrinth. Those in which the labyrinth was located lateral to the LSL were classified as lateral. Cases in which the LSL crossed the semicircular canal or crus commune were classified as medial (Figure 1).

### Surgical Procedure

Patients underwent CT using a mobile scanner (NL3000 CereTom®; Neurologica, Danvers, MA, USA) in the operating room with 8 to 10 markers affixed to the head. DICOM files were imported into the navigation system station. After automatic fusion of the CT and MRI sequences and their verification, the images were used for intraoperative navigation. All patients were placed in a semi-seated position for surgery. After the sigmoid and transverse sinuses were localized using the navigation system, a skin incision was designed. A 4 cm craniotomy was performed posterior to the sigmoid sinus and inferior to the transverse sinus. The dura was incised and the cerebellum retracted. After partial tumor debulking, the internal acoustic pore was exposed and the dura over the posterior lip of the IAC was removed. The extent of IAC removal was determined using intraoperative image guidance. The navigation optical pointer was used for positioning, and a projection of 5–10 mm was preset to represent the predicted resection line. The lead surgeon focused on the operation under a microscope, while another neurosurgeon acted as an assistant and made judgments based on the navigation and live video monitors. The upper, lower, and outer boundaries of drilling were identified. The outermost border of the tumor and the locations of the labyrinth and petrous cells were also considered. Image guidance was used repeatedly during drilling.

The posterior IAC wall was opened using a high-speed diamond drill under continuous saline irrigation. Drilling was considered adequate if the outermost tumor margin could be exposed by gently pulling the tumor. Regardless, drilling of the posterior wall of the IAC ceased when intraoperative image guidance indicated that only 2–3 mm of bone remained over the labyrinthine structures. Tumor dissection was performed along the plane between the tumor and capsule; the capsule close to the nerves was retained. After microsurgical tumor removal was accomplished, a 0-degree/30-degree endoscope was fixed with a mechanical arm and introduced into the surgical field to visualize areas of the surgical site that the microscope could not. If endoscopic examination revealed any remaining tumor, it was carefully dissected and removed. After complete resection was confirmed and hemostasis achieved, the IAC was packed with pieces of fat under endoscopic visualization. Patients were transferred to the neurological intensive care unit after surgery was completed.

### Definitions and Outcomes

The length of the IAC was measured on preoperative CT images and postoperative labyrinth integrity assessed by CT. The presence of residual tumor within the IAC was determined by endoscopic examination during surgery and MRI immediately after surgery. Additionally, postoperative facial nerve function was classified according to House–Brackmann (HB) grade, HB grades 1 or 2 being considered good whereas HB grade 3 and above were considered to denote facial nerve dysfunction. Hearing function was considered useful in patients with pure tone average better than 50 dB and speech discrimination score better than 50% according to Gardner-Robertson grading system. Postoperative CSF leakage, delayed hemorrhage and other complications were recorded.

### Statistical Analysis

Continuous variables are presented as means with standard deviation. Categorical variables are presented as frequencies with percentage. Comparisons were performed using the chi-square test, Fisher's exact test, or Student's t-test as appropriate. Statistical analyses were performed using SPSS software version 23.0 (IBM Corp., Armonk, NY, USA). *P* < 0.05 was considered significant.

## Results

### Patient and vs Characteristics

Data of 84 patients were analyzed. Microsurgical removal with navigation and endoscopic assistance was achieved in 53 patients (CS group), and microsurgical removal without such assistance in 31 (MS group). Patient characteristics according to group are shown in Table 1. There were 29 lateral tumors and 24 medial ones in the CS group. Patient characteristics according to type are shown in Table 2.

**Table 1:** Patient characteristics of two groups.

	MS group	CS group	*P*-value
No.	31	53	
Age (years)	51.7 ± 11.5	53.1 ± 10.5	0.58
Sex, M:F	11:20	20:33	>0.99
Side, R:L	11:20	32:21	0.04
Tumor size (mm)	32.6 ± 9.1	28.9 ± 8.6	0.07
Length of IAC (mm)	9.0 ± 1.5	9.3 ± 1.6	0.42
Tumor residue (postoperative MR)	5	1	0.02
Normal rate of FN	64.5%	81.1%	0.12
Normal rate of FN	80.6%	90.6%	0.31
Useful hearing (post-/pre-operative)	5/12	8/16	>0.99
Labyrinth injury	4	0	0.02
Opened the air cells	3	7	0.74
CSF leak	3	2	0.35
ICH	2	1	0.55

*M, male; F, female; R, right; L, left; IAC, internal auditory canal; CSF, cerebrospinal fluid.*

*ICH, intracranial hemorrhage; FN, facial nerve.*

**Table 2:** Patient characteristics between the two types.

	Medial type	Lateral type	*P*-value
No.	24	29	
Age(years)	53.3 ± 10.3	52.9 ± 10.9	0.89
Sex, M:F	12:12	8:21	0.15
Side, R:L	17:7	15:14	0.17
Tumor size(mm)	31.5 ± 9.0	27.0 ± 7.9	0.06
Length of IAC (mm)	8.9 ± 1.6	9.6 ± 1.6	0.10
Tumor residue (endoscope)	5	0	0.02
Tumor residue (postoperative MR)	1	0	0.45

*M, male; F, female; R, right; L, left; IAC, internal auditory canal.*

### Surgical Results

In the CS group, the fundus of the IAC was examined endoscopically after microsurgery, revealing and enabling removal of residual tumor tissue in five patients. Residual tumor was detected by postoperative MRI in one patient. In the MS group, postoperative MRI showed residual tumor at the fundus of the IAC in five patients. The rate of residual tumor at the fundus of the IAC was lower in the CS than in the MS group (*P* = 0.02). Postoperative facial nerve status and preservation of useful hearing are shown in Table 1; the differences between the two groups are not statistically significant. In the CS group, the incidence of endoscopic-assisted resection of residual tumor at the IAC was significantly higher for medial than for lateral tumors (20.8% vs. 0%, *P* = 0.02) (Table 2).

### Complications

The labyrinth was not damaged in any patient in the CS group, whereas labyrinthine injury occurred in four patients in the MS group, three of whom had no useful hearing preoperatively. Other complications are shown in Table 1.

### Representative Case

**Figure 2:**
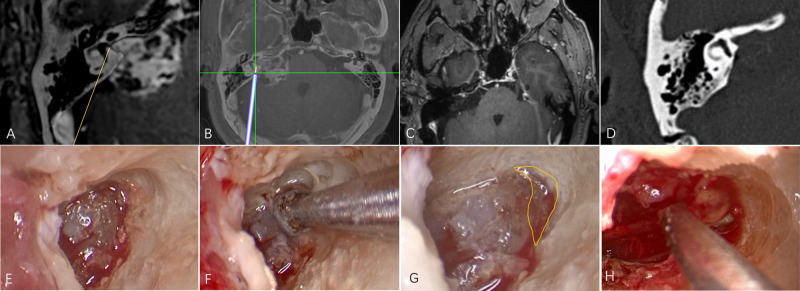
An illustrative case. (**A**) The tumor was classified as of medial type on the basis of fusion images. (**B**) Intraoperative navigation. (**C**) Postoperative MR showing that the tumor within the IAC has been completely resected. (**D**) Postoperative CT showing maximal removal of the posterior wall of the canal. (**E**) Endoscopic image after drilling under microscope guidance. (**F**) Endoscopic drilling of the deep bone; (**G**) The area within the yellow line shows the extent of endoscopic drilling. (**H**) Endoscopic resection of residual tumor at the fundus of the IAC.

A 56-year-old man with hearing loss and tinnitus was found to have a tumor in the right cerebellopontine angle by MRI. The tumor was classified as of medial type and excised with navigation and endoscopic assistance. Postoperative MR images showed that the tumor within the IAC had been totally resected. Postoperative CT showed that the posterior wall of IAC had been maximally drilled and the labyrinth remained intact (Figure 2).

## Discussion

The goal of VS surgery is complete resection while preserving neurological function, which is challenging (1, 4, 5, 25, 26). For large VSs, the RS approach is preferred when hearing preservation is required. Complete resection when performing the RS approach requires opening the IAC. It is essential to maintain the integrity of the labyrinth while drilling the posterior wall of the canal during this opening if hearing is to be preserved. However, because the local anatomy of the labyrinth, IAC, and tumor can vary widely, a gold standard for the angle or extent of drilling that will preserve hearing has not been established (3, 6, 10, 27–29). Therefore, it is necessary to determine the local anatomy before surgery.

Ciric et al. recommend removing no more than 8 to 9 mm of the posterior canal wall unless the posterior semicircular canal and/or endolymphatic duct are opened (30). Yokoyama et al. proposed the concept of the sigmoid–fundus line, an imaginary line extending from the medial side of the sigmoid sinus to the fundus of the IAC on CT (8). Samii et al. and Gharabaghi et al. concluded that if the posterior semicircular canal and crus commune are located lateral to this line, there is no risk of intraoperative injury to these structures; however, if they are located medial to the line, they are at risk (3, 9). CT does not provide a clear outline of the tumor. The purpose of posterior IAC wall drilling is to expose the lateral edge of the tumor, not the fundus of the IAC. Mohr et al. reported that approximately 60% of VSs do not completely fill the IAC (31). MRI can be used to determine the extent of tumor in the IAC, which seems to guide IAC drilling. However, MRI does not show the position of the semicircular canals because this imaging modality does not demonstrate bony structures very well. We discovered this with our use of CT/MRI fusion images for surgical navigation, which showed tumor, sigmoid sinus, posterior IAC wall, and internal IAC structures simultaneously. We were able to improve upon the sigmoid–fundus line using the fused images to define an imaginary line between the outermost edge of the tumor and the medial side of the sigmoid sinus as the LSL. The risk of Labyrinth injury can be assessed according to the relationship between the LSL and the posterior semicircular canal and crus commune before drilling.

**Figure 3:**
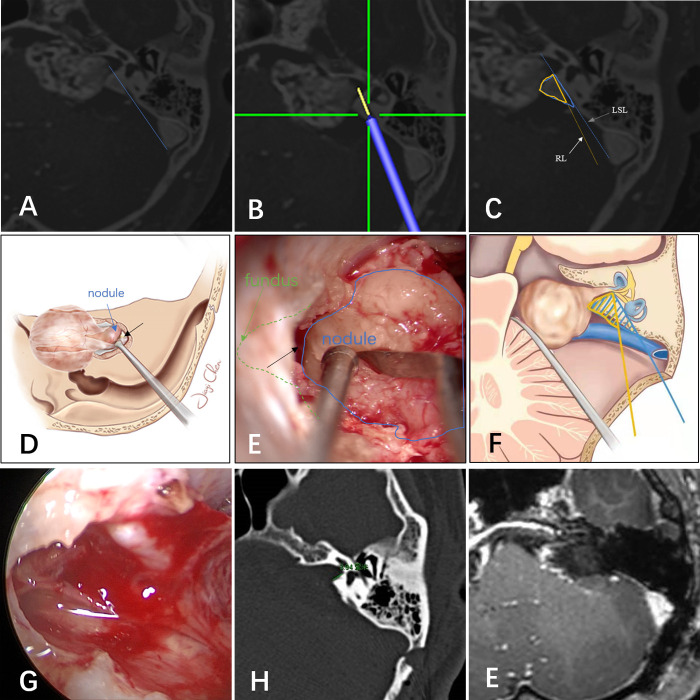
(**A**) The tumor was classified as lateral based on magnetic resonance imaging and computed tomography fusion images. (**B**) Navigation predicted the resection line. The yellow line extending from the tip of the navigation pointer is the predicted resection line. (**C**,**F**) Illustration of the lateral safe line (blue line) and resection line (yellow line). Illustrative drawing (**D**) and microscopic view (**E**): the intrameatal portion of the tumor is displayed as a nodule (blue). The interface (black arrow) between the tumor (blue) and fundus (green) was visible by gently pulling the tumor medially. This means that the opening of the IAC was adequate. (**G**) Endoscopic examination showed no residual tumor, and the capsule near the nerves was retained. Postoperative CT(**H**) and MR(**I**) showed no tumor residue and the labyrinth was intact.

It should be noted that the LSL is neither a surgical line of sight nor an IAC resection line. By retracting the cerebellum, the IAC can be drilled at a more medial angle than the LSL (8). However, it is not practical to precisely simulate the line of sight, because the amount of space gained by cerebellar retraction cannot be accurately assessed before surgery. In addition, the degree of traction required varies according to the patient and surgeons differ in their comfort level with applying traction. Figure 3 shows illustrative preoperative navigation imaging, intraoperative photography, and postoperative imaging in a patient with a VS classified as lateral. As shown in panel C, the blue line represents the LSL and the blue area indicates the maximum area estimated to be removed before surgery. In general, IAC drilling starts at the posterior edge of the internal acoustic pore and proceeds laterally until the outermost part of the tumor is exposed. Owing to cerebellar retraction, the actual range of drilling during surgery is less than the blue area and comprises the area in the yellow triangle. Provided that the labyrinth is not within this triangle, it should not be damaged. Therefore, in this and other tumors classified as lateral, the tumor within the IAC can be exposed safely. Illustrative drawing (Figure 3D) and microscopic view (Figure 3E) showed that the intrameatal portion of the tumor is displayed as a nodule (blue). The interface (black arrow) between the tumor (blue) and fundus (green) was visible by gently pulling the tumor medially. This means that the opening of the IAC was adequate. In these cases, no residual tumor was found during intraoperative endoscopic examination.

In medial cases, tumor exposure is likely inadequate because drilling must be stopped near the labyrinth. Marchioni et al. argue that exposure of the IAC fundus is incomplete when only the microscope is used during the RS approach and therefore a tumor located in the fundus requires blind dissection (15). Endoscopy is useful in the RS approach as an adjunct to microscopic resection and can even be implemented alone in fully endoscopic procedures (16). Shahinian et al. reported full endoscopic resection in a series of 527 patients with unilateral VS (13). You et al. recommended the use of endoscopic-assisted microsurgery (14). According to previous studies, the incidence of residual tumor detected by endoscopic examination after microscopic resection ranges between 6.5% and 48.1% (Table 3). In our study, the overall incidence of residual tumor in the blind corner was 9.4% and all instances were in cases classified as medial. No lateral cases had residual tumor detected in the endoscopic examination (20.8% vs. 0%; *P* = 0.015). This means that the intrameatal portion of VSs cannot be completely exposed and resected without damaging the labyrinth in one-fifth of medial cases. Therefore, we suggest that endoscopic-assisted resection should be performed for cases that are classified as medial on preoperative imaging (those in which the LSL crosses the semicircular canal or crus commune). CT/MRI fusion images assess not only the degree of tumor within the IAC, but also the position of the labyrinth relative to the tumor, which provides a more comprehensive and accurate assessment than either modality alone.

**Table 3:** Endoscope check the residual tumor.

Authors & Year	Approach	Endoscope (tip angle)	Cases	Endoscope Check Residual Tumor (%)
Wackym, 1999	RS	30°	68	11 (16.2%)
Göksu, 1999	RS-RL	0°,30°,70°	32	8 (25.0%)
Kumon, 2011	RS	30°,70°	27	13 (48.1%)
Chovanec, 2012	RS	0°,30°,70°	39	4 (10.2%)
Marchioni, 2019	RS	0°,45°,70°	18	4 (22.2%)
Bi, 2022	RS	0°,30°	61	4 (6.5%)

**Figure 4:**
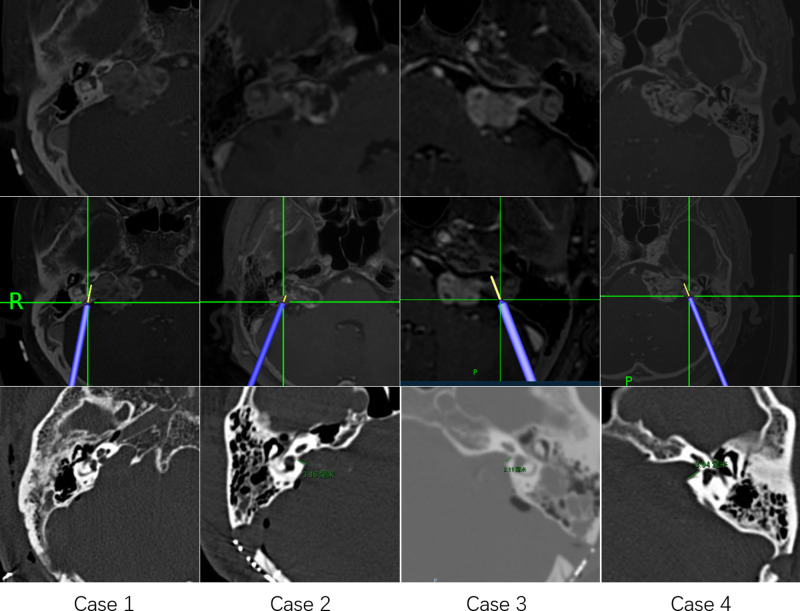
Four illustrative cases of using navigation to locate the boundaries of drilling of the internal auditory canal.

Pillai et al. concluded that approximately 70% to 80% of the posterior wall of the IAC can be drilled away without injuring the labyrinth and that such drilling can be maximized safely using image-guided surgical navigation (10). In the middle fossa approach to VSs, Miller et al. found surgical navigation to be reliable in identifying the midpoint of the IAC within 2.4 mm of accuracy (1). Furthermore, Li et al. reported localization of the facial nerve for surgical purposes using tractography and navigation (25). However, Samii et al. concluded in a preliminary clinical study that the value of navigation-assisted IAC opening is promising but limited at the present time (3, 9). Although surgical navigation has gained wide acceptance for many intracranial procedures, it is rarely used for localization of the IAC during VS surgery. There are three possible explanations. First, surgeons may have concern about navigation system accuracy. However, with the use of invasive localization markers and point-merge registration techniques, navigation can achieve sub-millimeter mean registration error (10, 32). In an accuracy study of surgical navigation in the spine, Devito et al. found that 98.3% of pedicle screws inserted using robotic guidance were either completely within the pedicle or breached it by 2 mm or less (33). Second, many experienced surgeons may believe that navigation offers limited benefits. Computer-aided navigational tools are no substitute for a thorough knowledge of temporal bone anatomy (1, 9). In our experience, VS surgery requires considerable experience to perform well. Navigation may be an imperfect but trustworthy partner in the learning process (Figure 4). When using it, a “safety zone” of approximately 2 mm should be set. Third, use of navigation can interrupt the operation (9). All types of surgical navigation require linking a virtual picture with the surgical field of view. Although current virtual reality technology is advancing, it is currently insufficient (34). Our solution is to perform the procedure using two surgeons, one focusing on the surgical field of vision and the navigation pointer, and the other focusing on the navigation display and the real-time picture of the operation. In addition, the cerebellum has been retracted when the navigation pointer was located in the posterior wall of the IAC, and the resection line (RL, the lateral edge of the IAC resection area, yellow line in Figures 3B,C) could be predicted by the navigation system. According to the RL line, the risk of labyrinth during drilling could be assessed again. If labyrinth structures were located 2 mm lateral of the RL line, repeated image guidance was not required during drilling the IAC.

CSF leakage is a common complication of VS surgery (35). Although the incidence is low, it often requires operative treatment if CSF leakage persists. Yamakami et al. state that packing of the opened IAC with pieces of fat and muscle is mandatory to prevent CSF rhinorrhea in patients with air cells near the canal (36). In addition, Tamura et al. and Corrivetti et al. argue that use of an endoscope can assist visualization and covering of opened petrosal air cells (16, 37). Endoscopic evaluation of the petrous bone may reduce the incidence of CSF leakage (17). In our study, the incidence of postoperative CSF leakage was 3.7% (2/53) in the CS group compared with 9.7% (3/31) in the MS group. This difference was not statistically significant; however, the use of navigation and endoscopic assistance in opening the IAC may nevertheless help to minimize CSF leakage. The hearing preservation is an important objective for the protection of labyrinth during surgery. However, the average diameter of the tumors in this study was close to 3 cm and most of them were Grade 3 or 4 according to the Koos classification. Only a few patients had useful hearing preoperatively, which greatly limited our analysis of hearing preservation.

## Conclusion

Navigation and endoscopy are useful in assisting the exposure of the intrameatal portion of VSs. Preoperative MRI/CT fusion imaging is helpful in preoperative evaluation and surgical planning in patients undergoing VS surgery. Tumors of the medial type require endoscopic assistance for resection.

## Data Availability

The raw data supporting the conclusions of this article will be made available by the authors, without undue reservation.
